# Combination of Hydroxychloroquine Plus Azithromycin As Potential Treatment for COVID-19 Patients: Safety Profile, Drug Interactions, and Management of Toxicity

**DOI:** 10.1089/mdr.2020.0232

**Published:** 2021-03-12

**Authors:** Guillaume Hache, Jean Marc Rolain, Philippe Gautret, Jean-Claude Deharo, Philippe Brouqui, Didier Raoult, Stéphane Honoré

**Affiliations:** ^1^ Service de Pharmacie, Hôpital de la Timone, APHM, Marseille, France.; ^2^ Aix Marseille Univ, INSERM, INRAE, C2VN, Marseille, France.; ^3^ IHU-Méditerranée Infection, Marseille, France.; ^4^ Aix Marseille Univ, IRD, APHM, MEPHI, Marseille, France.; ^5^ Aix Marseille Univ, IRD, AP-HM, SSA, VITROME, Marseille, France.; ^6^ Service de Cardiologie, Hôpital de la Timone, APHM, Marseille, France.; ^7^ Aix Marseille Univ, Laboratoire de Pharmacie Clinique, Marseille, France.; ^8^ Aix-Marseille Univ, CNRS, INP, Inst Neurophysiopathol, Marseille, France.

**Keywords:** hydroxychloroquine, azithromycin, COVID-19

## Abstract

The coronavirus disease 2019 (COVID-19), caused by infection with severe acute respiratory syndrome coronavirus 2, has recently emerged worldwide. In this context, there is an urgent need to identify safe and effective therapeutic strategies for treatment of such highly contagious disease. We recently reported promising results of combining hydroxychloroquine and azithromycin as an early treatment option. Although ongoing clinical trials are challenging the efficacy of this combination, many clinicians claim the authorization to or have already begun to use it to treat COVID-19 patients worldwide. The aim of this article is to share pharmacology considerations contributing to the rationale of this combination, and to provide safety information to prevent toxicity and drug–drug interactions, based on available evidence.

## Introduction

The coronavirus disease 2019 (COVID-19), caused by infection with severe acute respiratory syndrome coronavirus 2 (SARS-CoV-2), has recently emerged worldwide. In this context, there is an urgent need to identify safe and effective therapeutic strategies for treatment of this new and highly contagious disease. Such highly contagious outbreaks are usually very sudden and spread for a relative short period of time. Although the clinical development of new molecules is needed for postpandemic cases and potential epidemic recurrences, clinicians have to cope with the immediate problem according to best scientific knowledge and medical experience. As time required to conduct well-designed clinical trials is not compatible with the clinical management of the first wave of infection, the only available pharmacological tools are medications already approved for other diseases, candidate for a drug repositioning. Drug repositioning is thus an approach that is currently being pursued to identify safe and effective treatments against COVID-19.

In COVID-19 patients, the transition from the first symptoms of this viral infection to acute respiratory distress syndrome is likely due to uncontrolled cytokine release.^1^ Hydroxychloroquine, which is better tolerated than chloroquine in humans, exhibits both an antiviral activity against SARS-CoV-2 *in vitro* and strong immunomodulatory capacities that can be useful in COVID-19.^2^ Potential mechanisms of action of hydroxychloroquine in COVID-19 disease have been reviewed recently.^3^ In a single-center nonrandomized trial conducted at IHU-Méditerranée-Infection-APHM in which 24 positive COVID-19 patients were treated with hydroxychloroquine 600 mg per day for 10 days and antibiotic therapy if necessary (amoxicillin–clavulanic acid or azithromycin), 70% of patients who received hydroxychloroquine were PCR negative at day 6, compared with 12% for the controls. In addition, all patients who received hydroxychloroquine and azithromycin were tested negative on day 6, suggesting a potential benefit of the association as compared with amoxicillin–clavulanic acid.^4^ Although this study has been criticized for methodology and statistics, such as the lack of size estimates, a secondary analysis has been performed by an external nonrelated expert.^5^ The conclusion of this second statistical analysis was that the study does not provide sufficient evidence to support any effect of hydroxychloroquine monotherapy for treatment in COVID-19 and larger randomized studies should be considered. However, the data of Gautret *et al.* do suggest that the drug combination therapy associating hydroxychloroquine and azithromycin is of great interest in COVID-19 and should be prioritized as soon as possible.

Then, an observational study was also conducted at IHU-Mediterranee-Infection-APHM on 80 patients treated with hydroxychloroquine–azithromycin combination and has confirmed the previous observation.^6^ Indeed, a rapid fall of nasopharyngeal viral load tested by quantitative polymerase chain reaction was noted, with 83% negative at day 7 and 93% at day 8. Virus cultures from patient respiratory samples were negative in 98% patients at day 5 that is higher than the percentage observed in the literature.

From these observations, 58 trials involving hydroxychloroquine and/or azithromycin have been registered in clinicaltrials.gov so far (*April 13, 2020*) and 14 trials worldwide aim to evaluate the combination of both, with various dose regimen (Table 1). More recently, we provided the clinical and viral outcomes of our cohort of 1,061 COVID-19 patients, treated at least 3 days with the hydroxychloroquine–azithromycin combination.^7^ We described good clinical outcomes with virological cure obtained for 91.7% of the patients, and with prolonged viral carriage at completion of the treatment for 4.4% of them. Poor clinical outcomes were described for 4.3% of the patients, including five deaths (0.5%). These observations suggest that the combination is safe and may avoid worsening, virus persistence, and subsequent contagiosity.

**Table 1.** Ongoing Trials Investigating Hydroxychloroquine and Azithromycin in the Treatment of COVID-19

Identifier	Title	Country	Regimen
NCT04341870	Study of Immune Modulatory Drugs and Other Treatments in COVID-19 Patients: Sarilumab, Azithromycin, Hydroxychloroquine Trial—CORIMUNO-19—VIRO	France	HCQ: 600 mg per day for 10 days
			Azithromycin: 500 mg D1 then 250 mg per day for 4 days.
NCT04341727	Hydroxychloroquine, Azithromycin in the Treatment of SARS-CoV-2 Infection	United States	HCQ: 800 mg on D1, then 400 mg per day for 4 days
			Azithromycin: 500 mg D1 then 250 mg per day for 4 days.
NCT04341207	Epidemiology of SARS-CoV-2 and Mortality to COVID-19 Disease in French Cancer Patients	France	HCQ: 600 mg per day for 10 days
			Azithromycin: 500 mg D1 then 250 mg per day for 4 days.
NCT04339816	Azithromycn Added to Hydroxychloroquine in Patient Admitted to Intensive Care with COVID-19 Randomised Controlled Trial	Czech republic	HCQ: 800 mg on D1, then 400 mg per day for 4 days
			Azithromycin: 500 mg D1 then 250 mg per day for 4 days.
NCT04338698	Hydroxychloroquine, Oseltamivir and Azithromycin for the Treatment of COVID-19 Infection: A RCT	Pakistan	HCQ: 600 mg per day for 5 days
			Azithromycin: 500 mg D1 then 250 mg per day for 4 days.
NCT04336332	Randomized Comparison of Combination Azithromycin and Hydroxychloroquine vs. Hydroxychloroquine Alone for the Treatment of Confirmed COVID-19	United States	HCQ: 600 mg per day for 10 days
			Azithromycin: 500 mg D1 then 250 mg per day for 4 days.
NCT04335552	Pragmatic Factorial Trial of Hydroxychloroquine, Azithromycin, or Both for Treatment of Severe SARS-CoV-2 Infection	United States	HCQ: 800 mg on D1, then 600 mg per day for 4 days
			Azithromycin: 500 mg D1 then 250 mg per day for 4 days.
NCT04334512	A Study of Quintuple Therapy to Treat COVID-19 Infection	United States	*Dose regimen not specified*
			Length: 24 weeks
NCT04332094	Clinical Trial of Combined Use of Hydroxychloroquine Azithromycin and Tocilizumab for the Treatment of COVID-19	Spain	HCQ: 800 mg on D1, then 400 mg per day for 6 days
			Azithromycin: 500 mg per day for 3 days
NCT04329572	Efficacy and Safety of Hydroxychloroquine and Azithromycin for the Treatment of Hospitalized Patients with Moderate to Severe COVID-19	Brazil	HCQ: 800 mg on D1, then 400 mg per day for 4 days
			Azithromycin: 500 mg per day for 5 days
NCT04328272	Effectiveness of Hydroxychloroquine in COVID-19 Patients	Pakistan	HCQ: 1,200 mg on D1 then 400 mg per day for 6 days
			Azithromycin: 500 mg D1 then 250 mg per day for 6 days
NCT04322396	Proactive Prophylaxis with Azithromycin and Chloroquine in Hospitalized Patients	Denmark	*Dose regimen not specified*
			Length: 15 days
NCT04322123	Safety and Efficacy of Hydroxychloroquine Associated with Azithromycin in SARS-CoV-2 Virus (COVID-19)	Brazil	HCQ: 800 mg per day for 7 days
			Azithromycin: 500 mg per day for 7 days
NCT04321278	Safety and Efficacy of Hydroxychloroquine Associated with Azithromycin in SARS-CoV-2 Virus (Coalition Covid-19 Brasil II)	Brazil	HCQ: 800 mg per day for 10 days
			Azithromycin: 500 mg per day for 10 days

Consulted on April 13, 2020.

D1, day 1; HCQ, hydroxychloroquine.

A recent prospective randomized clinical trial confirmed that oral treatment with hydroxychloroquine 200 mg BID significantly decreased the time to reach viral RNA negativity and the time to clinical recovery.^8^ Notwithstanding, clearing the ability to detect the virus does not mean that morbidity and mortality from virus are decreased. For example, in recent observational studies including in-hospital patients, patients treated with hydroxychloroquine showed no difference regarding risk of death or intubation compared with patients under other treatments.^9,10^ However, the patients included in the group receiving hydroxychloroquine, or hydroxychloroquine plus azithromycin had more severe disease and had more comorbidities. Conversely in large retrospective studies, hydroxychloroquine plus azithromycin treatment was associated with (1) a shorter duration of viral shedding and a decreased risk of transfer to ICU or mortality in ambulatory patients^11^ and (2) a decreased mortality in hospitalized patients.^12^ We have recently carried out a meta-analysis of all the work done on hydroxychloroquine in the context of COVID-19, focusing on mortality and viral shedding.^13,upgraded in 14^ Interestingly, hydroxychloroquine therapy was significantly and reproducibly correlated with a twofold decrease in both mortality and viral shedding.

Whereas ongoing clinical trials are challenging the efficacy of this combination, many clinicians claim the authorization to or have already begun to use it to treat COVID-19 patients worldwide. Thus, the aim of this article is to describe pharmacology considerations contributing to the rationale of this combination, and to provide safety information to prevent toxicity and drug–drug interactions, based on available evidence.

## Presentation of Medications

### Hydroxychloroquine

#### Description

Hydroxychloroquine is a drug originally used since 1952 in the treatment of malaria in the form of hydroxychloroquine sulfate. Its use as an antimalarial has greatly decreased due to the development of resistance in *Plasmodium*, the parasite responsible for malaria. The current use is mainly focused in rheumatology, for the treatment of rheumatoid arthritis and systemic lupus erythematosus due to its anti-inflammatory and immunomodulatory properties.

#### Pharmacokinetics of hydroxychloroquine

Hydroxychloroquine is well absorbed within 2 to 4 hr when given orally in doses from 100 to 1,200 mg daily and the fraction absorbed is estimated to be ∼75%.^15^ Hydroxychloroquine binds to both albumin and alpha-glycoprotein (50%) and blood concentration peaks shortly after the absorption phase and falls relatively quickly due to rapid partitioning into organs. Indeed, the accumulation of hydroxychloroquine in lysosomes appears to drive the large volume of distribution in plasma, whereas binding to melanin contributes to the long terminal half-life that is estimated between 30 and 50 days.^16^ The metabolism of hydroxychloroquine has been extensively studied in humans. Sixty percent is excreted unchanged in urine and the remaining is dealkylated by cytochrome P450 (CYP) to pharmacological active monohydroxylhydroxychloroquine and *N*-desethylhydroxychloroquine (16% and 18%, respectively).^16–18^ Thus, hydroxychloroquine and metabolites are renally excreted and consequently renal impairment is likely to increase the circulating concentration of the drug and risk of toxicity. In a large study evaluating the systemic factors that determine serum concentration of hydroxychloroquine, renal failure was associated with a significantly higher serum hydroxychloroquine concentration. Moreover, no association of ethnicity or smoking with blood hydroxychloroquine concentrations was found.^19^

#### Daily dose of hydroxychloroquine

*In vitro*, hydroxychloroquine had antiviral activity and decreased viral replication in a concentration-dependent manner. The EC_50_ values were 6.14 and 0.72 μ million (M) at 24 and 48 hr, respectively.^20^ As a matter of fact, hydroxychloroquine plasma levels were >0.5 μM from 30 min to 24 hr after a single administration of 400 mg of hydroxychloroquine (C_max_ = 1.2 ± 0.4 μM).^17^ Our preliminary results reported serum concentrations ∼0.75 μM at day 2 after 600 mg per day.^6^ Interestingly, a recent translational pharmacokinetic/pharmacodynamic (PK/PD) model suggested an optimized hydroxychloroquine dosing between >400 mg BID for >5 days to ensure the highest likelihood of success as a COVID-19 treatment.^21^ However, some authors suggested that plasma levels are unlikely to reflect the biodistribution of hydroxychloroquine, particularly in lungs. Indeed, lung accumulation is mainly driven by macrophages, which are not the main target of the virus.^22^ Thus, the authors suggested that whole blood concentrations may be more relevant, especially because it includes drug entrapped in lysosomes. Their mathematical model estimated that the administration of 600 mg per day may lead to drug levels of 6.16 μM in the whole blood.^23^

Related to safety, different daily doses of hydroxychloroquine have been investigated in randomized double-blind clinical trials for a period of 6 weeks in patients treated for rheumatoid arthritis.^24^ Dose levels tested were 400, 800, and 1,200 mg per day. Discontinuations of treatment due to adverse events appeared dose related (4% at 400 mg per day, 7% at 800 mg per day, and 9% at 1,200 mg per day) without any statistical difference between the three groups of patients. Reasons for discontinuations were mostly for gastrointestinal symptoms. The only significant difference between the three groups was for nausea and vomiting occurrence. Overall it seems that toxicity of hydroxychloroquine was not dose related (in this range of doses) and dose up to 1,200 mg per day for 6 weeks was well tolerated except for the gastrointestinal tract. Recently, some studies reported harm related to hydroxychloroquine administration to treat COVID-19 patients.^25,26^ The outcomes U.S. veterans treated by hydroxychloroquine suggest a mortality rate two to three times higher than no hydroxychloroquine. It is rather impossible to attribute the causality to hydroxychloroquine since pretreatment lymphopenia was two to three times higher in patients treated with hydroxychloroquine. Lymphopenia is a predictive factor of disease severity and death in COVID-19.^27^

Interestingly, a phase IIb clinical trial including 120 COVID-19 patients illustrates that a dose regimen of 1,200 mg per day during 10 days increased the risk of QT prolongation compared with 900 mg per day during 4 days.^26^ Electrocardiographic abnormalities developed during hospitalization for COVID-19 pneumonia were described in a specific study and reflect a wide spectrum of cardiovascular complication, including rhythm disorders.^28^ In this study, the authors identified that treatment with antiretroviral and hydroxychloroquine was associated with a significant reduction in the risk of developing new electrocardiogram (ECG) abnormalities in hospitalized patients. These results suggest that ECG abnormalities are part of the COVID-19 syndrome and may have a clinical impact on the course of the disease. Moreover, ophthalmologic adverse events were not dose related in this study. In fact, retinal toxicity prevalence is 8% in patients taking hydroxychloroquine for >5 years, rising to ∼20% after 20 years of treatment.^29^ Risk factors for this retinal toxicity are treatment duration (>5 years), daily dose (>5 mg/kg per day based on actual body weight), and cumulative dose >1,000 g.^29^ Thus, short treatment may not lead to retinopathy. Finally, antimalarial agents are associated with oxidative hemolysis, especially in patients with severe variants of glucose-6-phosphate dehydrogenase deficiency. However, such events were not reported in a survey of 275 rheumatology patients treated with hydroxychloroquine, with established G6PD deficiency.^30^ In summary, 600 mg per day is a dose regimen in the range of potential most effective regimen, while considering drug safety.

### Azithromycin

#### Description

Azithromycin is an azalide and is structurally related to the macrolide family of antibiotics. Macrolides have received considerable attention for their anti-inflammatory and immunomodulatory actions beyond the antibacterial effect. These two properties may ensure some efficacy in a wide spectrum of respiratory viral infections, for review see Min and Jang.^31^

#### Pharmacokinetics of azithromycin

After oral administration, azithromycin is widely distributed in tissues and particularly in lung with an apparent steady-state volume of distribution of 31.1 L/kg. The major route of elimination is biliary excretion of azithromycin as unchanged drug, and 6% of administered dose is found as unchanged drug in urine over 1 week after administration. Terminal elimination half-life is estimated ∼68 hr.^32^

#### Dose of azithromycin

In total, 500 mg on the first day, then 250 mg in the morning from day 2 to day 5 is in accordance with the marketing authorization for superinfections of acute bronchitis. Recently, our team at IHU-Méditerranée-Infection demonstrated that the combination of hydroxychloroquine and azithromycin had a synergistic effect *in vitro* on SARS-CoV-2, at concentrations compatible with that obtained in humans.^33^ This synergistic effect on viral load has been illustrated in a small trial involving COVID-19 patients.^4^

## Pharmacological Considerations for Combination of Hydroxychloroquine and Azithromycin

Based on our preliminary results suggesting a potential benefit of the association to reduce viral load of SARS-CoV-2, Sandeep and McGregor performed energetic-based modeling of hydroxychloroquine and azithromycin binding to the SARS-CoV-2 spike (S) protein–ACE2 complex.^4,34^ Binding experiments suggested that hydroxychloroquine may be ineffective in directly inhibiting the SARS-CoV-2 spike–ACE2 interaction by itself. Instead, it seems to increase the acidity of the ACE2 system in the interaction between the ACE2 and SARS-CoV-2 spike that could result in the degradation of the spike, and potentially to decrease virus dissemination. In contrast, azithromycin provides high binding affinity and may directly target the binding interaction point between the SARS-CoV-2 spike and ACE2.^34^ This may be responsible for the apparent synergism between hydroxychloroquine and azithromycin that we observed.^4,5^ These data give a pharmacological rationale for using this association in the context of COVID-19.

In contrast, some reports described ineffectiveness of macrolides in some viral infections such as rhinovirus infections,^35^ Middle East respiratory syndrome,^36^ and H1N1 patients,^37^ and even detrimental effect of hydroxychloroquine in some viral infections such as chikungunya virus and HIV.^38,39^ The suspected mechanism was that hydroxychloroquine inhibits the effect of T cells by interfering on interleukin 2 production,^40^ fundamental in priming and maintaining T-helper 2 cell response.^41^ Azithromycin may also exert immunomodulatory effects by inhibiting T-helper 2 cytokines.^42^ Our report on 1,061 COVID-19–treated patients did not unveil such detrimental effect.^7^

## Hydroxychloroquine–Azithromycin Contraindications

Contraindications for this combination are those reported for each individual drug and presented in Table 2.^43^

**Table 2.** Main Contraindications of Both Hydroxychloroquine and Azithromycin

	Hydroxychloroquine	Azithromycine
Absolute contraindications	Hypersensitivity to active substances (cross-reactivity): hydroxychloroquine or chloroquine, amino-4 quinoleines, amodiaquine, mefloquine, glafenine, floctafenine, antrafenine. Increased risk of cardiac arrhythmia: citalopram, escitalopram, hydroxyzine, domperidone, and piperaquine. Pathophysiology: retinopathy, age <6 years, weight <35 kg.	Hypersensitivity to active substances (cross-reactivity): azithromycin, erythromycin, chlarithromycin, dirithromycin, josamycin, midecamycin diacetate, roxithromycin, telithromycin, macrolides, ketolides, everolimus, pimecrolimus, sirolimus, temsirolimus, fidaxomicine. Hypersensitivity to excipient: peanut oil and soy. Hypersensitivity to lactose, abnormality of galactose metabolism, lactase deficiency, and digestive malabsorption/intolerance syndrome because of the presence of lactose as excipient. Pathophysiology: pseudomembranous colitis, anaphylactic shock, severe skin involvement, acute exanthematic pustulosis, DRESS syndrome, severe liver failure, patient taking colchicine, cisapride, dihydroergotamine, and ergotamine.
Relative contraindication or not recommended	Hepatic porphyria, lactation, G6PD deficiency, psoriasis, hypersensitivity to lactose, abnormality of galactose metabolism, lactase deficiency, and digestive malabsorption/intolerance syndrome because of the presence of lactose as excipient.	Cholestase, colchicine, cisapride, dihydroergotamine, and ergotamine.

DRESS, drug reaction with eosinophilia and systemic symptoms.

## Association of Hydroxychloroquine and Azithromycin, Drug–Drug Interaction, and Cardiac Toxicity

Both medications have been widely used over the past decades. For example, in the United States, 52.5M prescriptions were written for azithromycin in 2012 and 4.5M for hydroxychloroquine in 2013 according to IMS health. To date, there are very limited data about safety of this combination in COVID-19 patients. Thus, we initially assessed the safety of the protocol indirectly, in regard with the available literature about toxicity for each medication in their respective therapeutic areas, not given in association. Azithromycin and chloroquine do not exhibit any direct pharmacokinetic interaction.^44^ In addition, azithromycin plus chloroquine combination therapy was well tolerated for the treatment of uncomplicated *Plasmodium falciparum* malaria in two multicountry randomized clinical trials in African adults and can be used safely during pregnancy.^45,46^ The European Respiratory Society chILD guidelines recommend the use of either hydroxychloroquine or azithromycin as second line to treat childhood interstitial lung disease, and side effects and monitoring are very well documented.^47,48^

Chloroquine is known to increase the risk of prolongation of the QTc interval.^49^ A recent systematic review of the literature has investigated the cardiac complications attributed to hydroxychloroquine and chloroquine.^50^ Only 127 reported cases were identified and for most of them they were treated for long time treatment with a median of 7 years (3 days–35 years) and for a very high cumulative dose. The median cumulative dose was 1,235 g for hydroxychloroquine and 803 g for chloroquine, much higher than the cumulative dose in our protocol for hydroxychloroquine (*i.e.*, 6 g).^50^ Recently, a translational PK/PD model suggested that dose regimen >1,200 mg day may predict to prolong QTc intervals.^21^ A risk of QTc prolongation also exists for azithromycin, particularly for the elderly aged 60–79 years.^51^ Thus, clinical and electrocardiographic monitoring is recommended when prescribing the association.^52^

In addition, a large cohort study, which includes ∼1 million patients, did not identify any increased risk of severe adverse events at 30 day with hydroxychloroquine compared with sulfasalazine in patients with rheumatoid arthritis.^53^ However, in patients treated with hydroxychloroquine, their meta-analysis reported 1.8 cardiovascular-related deaths per 1,000 patients cotreated with azithromycin compared with 0.7 per 1,000 patients cotreated with amoxicilline. The authors concluded that the addition of azithromycin may induce cardiovascular mortality, potentially due to synergistic effect on QT prolongation. These conclusions should be taken with caution.

First, the prevalence of acute respiratory disease appeared higher in the hydroxychloroquine plus azithromycin group, suggesting that patients were more exposed to cardiac events related to respiratory disease. For example, myocarditis was reported in pulmonary infections, including COVID-19, and may cause sudden death.^54,55^ Based on our experience including more than three thousand COVID-19 patients, we reported one COVID-19-related myocarditis leading to death: a woman, 28 years old, neither treated with hydroxychloroquine nor with azithromycin. Second, the exposition to treatment in terms of dosing and length was not documented, which does not allow to estimate the contribution to dose-dependent toxicities. And last but not least, there may be a confusing message about a putative “synergistic” QT prolongation, which might suggest an increased mortality rate related to proarrhythmic effect. As a matter of fact, there was a small absolute increase in cardiovascular mortality after 5 days of azithromycin in an observational cohort^56^ and azithromycin was associated with a mild and dose-dependent prolongation of QT interval in healthy subjects treated with the combination of azithromycin and chloroquine, compared with chloroquine alone.^57^ The elevation reported for chloroquine–azithromycin in this study was in the same range than for azithromycin alone.^58^

It is important to note that most of the drug-induced QT prolongations result from blocking hERG potassium canal, which slows cardiac repolarization and may lead to sudden death. However, azithromycin has a low affinity for the hERG channel,^59^ and preclinical studies demonstrated that excessive doses of azithromycin failed to cause *torsade de pointes*.^60^ Instead of slowing repolarization, supratherapeutic dose of azithromycin prolonged the potential of action itself.^61^ The molecular mechanism underlying this unusual proarrythmic observation describes multi-ion channel blocking, increasing cardiac Na^+^ current to promote intracellular Na^+^ loading associated with intracellular Ca^2+^ overload.^62^ These observations may explain overdose-related polymorphic ventricular tachycardia without QT prolongation, and the authors speculate that concomitant block of cardiac Ca^2+^ current by azithromycin might also provide an additional potential protective mechanism to prevent early after depolarizations and torsades.

Specifically in COVID-19 patients, recent reports including 84 and 251 patients described a major QT prolongation (>500 ms) in 11% and 23% of patients with SARS-CoV-2 infection treated with hydroxychloroquine and azithromycin, respectively.^63,64^ One patient developed a polymorphic ventricular tachycardia suspected to be *torsade de pointe*. Similar trends are described in a cohort study of 90 hospitalized patients.^65^ In this study, the authors reported one case of *torsade de pointes* illustrating cardiotoxic risk of the combination in the context of COVID-19. Unfortunately, very less information about prior clinical conditions, disease severity, nor concomitant treatment specifically for this patient is available. It is suggested that the proportion of patients with COVID-19 developing extreme QT interval prolongation with the combination can be explained by the specific characteristics of the population of the study, which included older age, higher prevalence of underlying and acute renal failure, and coadministration of additional QT-prolonging medications, particularly amiodarone.^64^ Recommendations emphasize the importance of discontinuing all QT-prolonging drugs at the onset of combination therapy. For example in a chloroquine-related *torsade de pointe* described in an 84-year-old women with COVID-19, her medical history, including metastatic breast cancer, previous pulmonary embolism, and high blood pressure, illustrated her frailty, and memantine was discontinued when QT prolongation unveiled.^66^ Interestingly, in our cohort of 3,119 patients treated with the combination, QTc prolongation (>60 ms) was observed in 0.67% and no *torsade de pointe*.^11^

## Potential Drug–Drug Interactions with Concomitant Treatments

Potential drug–drug interactions may occur between hydroxychloroquine–azithromycin combination and other ongoing treatments, by either pharmacokinetic interactions or additive/synergistic toxicity.

### Pharmacokinetic interactions

The main CYP isoform involved in the metabolism of hydroxychloroquine is CYP3A4. Thus hydroxychloroquine may be substrate of kinetic interactions with inducers and inhibitors of CYP3A4. However, regarding the complex pharmacokinetic profile including active metabolites, and long half-life of hydroxychloroquine, it is rather unlikely that these interactions may have relevant clinical consequences with the short-term treatment. Also, it has been postulated that proton pump inhibitors, such as omeprazole and pantoprazole, may affect the activity of hydroxychloroquine, raising the possibility that oral bioavailability of hydroxychloroquine may be affected by the changes in intragastric pH.^67^ Similarly, azithromycine does not illustrate opportunity for clinically relevant pharmacokinetic drug–drug interactions.^68^

Hydroxychloroquine and azithromycin may interfere with the metabolism of other common drugs. For example, hydroxychloroquine interacts with metoprolol as a consequence of the effects of both of these on CYP2D6.^69^ This leads to increased maximal plasma concentrations (C_max_) and thus the bioavailability of metoprolol.

The effect of hydroxychloroquine in causing increased levels of digoxin has been noted and this is clearly an important drug interaction that may result in increased cardiotoxicity of digoxin when used in combination with hydroxychloroquine (RxFiles Detailing Program 2008). Another significant drug interaction of relevance is the wide spread use of antimalarials with methotrexate (MTX).^70,71^ Indeed, coadministration of hydroxychloroquine with MTX causes reduced C_max_ and increased T_max_. It has been suggested that this effect of hydroxychloroquine on the PK of MTX may explain the decrease of acute liver effects due to MTX.^72,73^

With regard to azithromycin drug–drug interactions, azithromycin is a weak inhibitor of CYP3A4. However, no clinically relevant interaction has been highlighted so far.^68^ In addition, it should be noted that azithromycin is a potent inhibitor of the drug transporter P glycoprotein and some reports have suggested that azithromycin may rarely increase the blood concentration of cyclosporine.^74–76^ Caution and therapeutic drug monitoring of cyclosporine are thus recommended during coadministration of azithromycin and cyclosporine. Caution may also be applied with other Pgp substrates, especially if their therapeutic index is narrow; for example, immunosuppressive tacrolimus, everolimus, and anticoagulant such as apixaban and dabigatran.

### Potential synergistic toxicity

Most of drug–drug interactions potentially associated with synergistic toxicity with concomitant treatments are related to risk of QT prolongation. They may increase the risk of cardiac arrhythmia and *torsade de pointe*.^77^ Thus, precaution has to be taken with other treatments at risk of QT prolongation. For example, antidepressants such as citalopram or escitalopram are contraindicated and tricyclic should be used with caution. Most antipsychotic drugs are also at risk of QT prolongation, including antidopaminergic antiemetics such as domperidone and metoclopramide. Importantly unbalanced electrolytes, such as magnesium and potassium, may increase the risk of cardiac arrhythmia, and hypomagnesemia and hypokaliemia are common in SARS-CoV-2 infection.^78^ Thus, potential cardiac toxicity of hydroxychloroquine and azithromycin can be prevented by a systematic ECG and ionogram analysis. In addition, pathogenic effect of COVID-19 can be expected, potentially contributing to disease outcome.^79^ In this objective, Canadian Heart Rhythm Societies recommend that the risk of drug proarrhythmia may be minimized during the pandemia by (1) discontinuing unnecessary medications that may also increase the QT interval, (2) identifying outpatients who are likely at low risk and do not need further testing (no history of prolonged QT, unexplained syncope or family history of premature sudden cardiac death, no medications that may prolong the QT interval, and/or prior known normal QTc), and (3) performing baseline testing in hospitalized patients or those who may be at higher risk. If baseline ECG testing reveals a moderately prolonged QTc, optimization of medications and electrolytes may permit therapy. If the QTc is markedly prolonged, drugs that further prolong it should be avoided, or expert consultation may permit administration with mitigating precautions.^80^

In addition, both the American College of Cardiology^81^ and the European Society of Cardiology^82^ have defined recommendations for the use of azithromycin and hydroxychloroquine in combination for COVID-19, complementary to performing ECG and avoidance of other QTc interval–prolonging agents whenever feasible. In patients who start combination therapy with azithromycin and hydroxychloroquine, they recommended (1) a careful evaluation of the patient's clinical features, especially elderly people and significant underlying cardiomyopathy or cardiac symptoms, (2) correction of hypokalemia to a level >4 mEq/L and of hypomagnesemia to a level of >2 mg/dL, and (3) discontinuation of potentially inappropriate therapy with proton pump inhibitors^83^ because they notoriously reduce the absorption of potassium and magnesium. Bun *et al.*^84^ showed that the combined therapy could be administered in more that 94% of inpatients who presented lower respiratory tract infection caused by SARS-CoV-2 after respecting these guidelines.

Besides this well-known cardiac toxicity, sparse reports described other kinds of nonexpected toxicities that might be cited to prevent any toxicity addition when used with other medications. For example, hydroxychloroquine may exert anticoagulant effect if high doses are prescribed.^85^ Some case reports described hemorrhage in patients treated with hydroxychloroquine for rheumatic diseases without being able to input causality relationship.^86–88^ Although hydroxychloroquine causality was difficult to document, the use of hydroxychloroquine may increase bleeding risk of patients undergoing anticoagulant therapy. In addition, case reports have described neuromyotoxicity and were consistent with studies highlighting the blockage of neuromuscular junction by hydroxychloroquine.^89,90^ These cases suggest that hydroxychloroquine may have synergistic effects and enhance curare action.^91^ Hydroxychloroquine can lower the convulsive threshold and there are some reports of seizure unveiled under hydroxychloroquine therapy.^92^ Coadministration of hydroxychloroquine with other drugs known to lower the convulsion threshold may increase the risk of convulsions. Also, the activity of antiepileptic drugs might be impaired if coadministered with hydroxychloroquine. Finally, some studies showed that treatment with hydroxychloroquine for a period of 6 months can effectively decrease blood glucose level and also hemoglobin A1c probably due to increased insulin production and secretion from B cells, or to decreased insulin clearance.^93^ There is no report of such hypoglycemic effect with short treatment with hydroxychloroquine, but it may be taken into consideration for patients with antidiabetic treatment.

## Conclusion

According to these data, the combination of hydroxychloroquine and azithromycin appears to have a theoretical safe profile with few clinically relevant drug–drug interactions. The main side effects related to hydroxychloroquine are gastrointestinal symptoms and mainly nausea, vomiting, and diarrhea. The main risk of the drug combination remains the risk of cardiac toxicity that can be prevented by respecting the contraindications of each drug, managing polypharmacy especially QT-prolonging concomitant medication, and monitoring through systematic electrocardiogram and ionogram during the association. Our current observations and practices illustrate the efficacy of risk management. Data about safety of an alternative dose regimen of this protocol, ongoing in clinical trials with COVID-19 patients, are needed.
